# Quality evaluation of *Alpinia oxyphylla* after *Aspergillus flavus* infection for storage conditions optimization

**DOI:** 10.1186/s13568-017-0450-x

**Published:** 2017-07-11

**Authors:** Xiangsheng Zhao, Jianhe Wei, Yakui Zhou, Weijun Kong, Meihua Yang

**Affiliations:** 1 Hainan Branch Institute of Medicinal Plant Development, Chinese Academy of Medical Sciences & Peking Union Medical College, Haikou, 571100 China; 2 Institute of Medicinal Plant Development, Chinese Academy of Medical Sciences & Peking Union Medical College, Beijing, 100193 China

**Keywords:** *Alpinia oxyphylla*, *Aspergillus flavus*, Aflatoxins, Storage conditions, Quality evaluation

## Abstract

In the storage of *Alpinia oxyphylla*, growth of mildew (especially toxic fungi, such as *Aspergillus flavus*) is a potential safety risk. Few reports have investigated how *A. oxyphylla* storage conditions impact mold growth or how mold growth impacts the bioactive components of *A. oxyphylla*. In this study, sterilized *A. oxyphylla* samples were contaminated by artificial inoculation of *A. flavus* spores. The main chemical components and aflatoxin levels in the infected *A. oxyphylla* samples were characterized. Central composite design-response surface methodology was used to study the effects of different temperature and humidity of storage conditions on the fungal growth in *A. oxyphylla* and accumulation of aflatoxins. The results showed that aflatoxins levels can be minimized by storing samples at temperatures below 25 °C and with humidity less than 85%. Additionally, we found that the yield and composition of volatile oil in *A. oxyphylla* exhibited small changes due to mold growth. However, polysaccharide content reduced remarkably. Temperatures below 25 °C and humidity below 85% were the best storage conditions to preventing *A. oxyphylla* from becoming moldy. Our results provide the theoretical basis for future studies the effects of storage conditions and mold growth on the quality and safety of traditional Chinese medicines (TCMs).

**Electronic supplementary material:**

The online version of this article (doi:10.1186/s13568-017-0450-x) contains supplementary material, which is available to authorized users.

## Introduction

Fungal contamination of medicinal plants can occur at any stage, from cultivation to sale in locations throughout the world. In tropical and subtropical areas in particular (Zhang et al. 2012), high temperatures and increased humidity are conducive for fungal growth and mycotoxin production. Mildew can degrade or destroy the active ingredients and produce toxic compounds, which reduces the efficacy of medicinal plants, and influences the quality and safety of their final products (Wang et al. 2015). *Aspergillus* and *Fusarium* fungi, including *Aspergillus flavus,* have been reported as the most common species of fungi in medicinal plants (Battilani et al. 2016). These species are of special concern because they can produce mycotoxins [e.g. aflatoxins (AFs), ochratoxin A] that pose potentially serious threats to human and animal health, due to their acute and chronic toxicity (Qin and Guo 2011). Hence, in recent years, fungal contamination, together with mycotoxin production, in medicinal plants has garnered growing attention.

*Alpinia oxyphylla* (Yizhi in Chinese, *A. oxyphylla*), the dried ripe fruits of *Alpinia oxyphylla* Miq, is a folk medicine with broad anti-inflammatory, anti-allergy, anti-ulcer and neuroprotective effects that can be used to treat intestinal disorders, diuresis, ulceration, dementia and other diseases (Li et al. 2013). In addition, *A. oxyphylla* is also consumed as a food in China. Phytochemical studies have indicated that it contains volatile oils, polysaccharides, flavonoids and diarylheptanoids (Li et al. 2013; Feng et al. 2015; Zhao et al. 2013). Because it is widely distributed in tropical and subtropical regions, *A. oxyphylla* can become moldy during its growth, harvesting, processing and storage. Environmental conditions, such as high temperature and humidity, are crucial for fungal contamination and mycotoxin production (Amelin et al. 2013). It has been reported that AFs contaminate up to 100% (*n* = 3) of *A. oxyphylla* samples, with maximum contamination levels of 0.81 μg/kg for AFB_1_ and 10.3 μg/kg for all AFs (the sum of AFB_1_, AFB_2_, AFG_1_ and AFG_2_) (Zha et al. 2014). Another report found that *A. oxyphylla* contained AFB_1_ at levels (up to 20 μg/kg) higher than the maximum residue limits established by the Chinese Pharmacopoeia (Zhang et al. 2008; Chinese Pharmacopoeia Commission 2015). These results suggested that *A. oxyphylla* might be infected by some species of *Aspergillus* during storage. Additionally, the authors observed that *A. oxyphylla* was commonly found to be moldy under improper storage conditions. However, as far as we know, research into the effects of storage conditions on *A. oxyphylla* quality and mold contamination has not been reported.

Hence, the aims of the present study were to (1) determine the ideal storage conditions (including temperature and humidity) for *A. oxyphylla* to prevent *A. flavus* infection and (2) to evaluate the impact of mold growth on *A. oxyphylla* quality. Using a trans-culturing approach, sterilized *A. oxyphylla* was artificially inoculated with *A. flavus* spores for cultivation. Central composite design-response surface methodology was used to study the effects of different storage temperature and humidity conditions on fungal growth in *A. oxyphylla* and subsequent production of aflatoxins. The volatile compound and polysaccharide composition was then determined by gas chromatography–mass spectrometry (GC–MS) and spectrophotometry, respectively. Aflatoxins (including AFB_1_, AFB_2_, AFG_1_ and AFG_2_) were detected by ultra-performance liquid chromatography coupled with tandem mass spectrometry (UPLC-MS/MS). This is the first study to optimize the storage conditions for *A. oxyphylla*, which will provide the theoretical basis to establish the most effective system to protect *A. oxyphylla* from mold infection during its storage.

## Materials and methods

### Chemicals, reagents and materials

Methanol, acetonitrile and ethyl acetate (Merck, Darmstadt, Germany) were of HPLC grade. All other chemical solvents of analytical grade were obtained from Beijing Chemical Reagents Co. (Beijing, China). Deionized water was prepared using a Milli-Q water purification system (Millipore Corporation, USA). Reference standards for AFB_1_, AFB_2_, AFG_1_, AFG_2_ were bought from Solarbio (Beijing, China). Nootkatone was obtained from Sigma-Aldrich Co. Ltc. (Shanghai, China). The purities of all the above reference compounds were above 98%. *Aspergillus flavus* strains were supplied by China General Microbiological Culture Collection Center (CGMCC 3.4410, Beijing, China). Aflatoxin-free *A. oxyphylla* samples were collected from Wuzhishan city, Hainan province, China.

### Instrumentation

UPLC coupled with a QTRAP 6500 triple quadruple mass spectrometer (AB Sciex, Toronto, ON, Canada) with electrospray ionization (ESI) was used to analyze *A. oxyphylla* samples. Chromatographic separation of four aflatoxins was performed on an Acquity BEH C_18_ column (100 mm × 2.1 mm i.d. 1.7 μm, Waters Corp., Milford, MA, USA). The mobile phase consisted of 0.2 mM ammonium acetate water (A) and MeOH containing 0.1% formic acid (B) at a flow rate of 0.3 mL/min. A gradient program was used as follows: 0 min: 25% A; 2 min: 45% A; 10 min: 90% A; 12 min: 90% A; 12.1 min: 25% A. The injection volume was 2 μL and the column temperature was set to 35 °C. ESI–MS/MS analyses were performed in positive ionization mode. The ionization source parameters were as follows: 550 °C, curtain gas (nitrogen), 35 psi; ion spray voltage 5000 V in positive mode; Gas 1 60 psi and Gas 2 55 psi. Data were acquired using the multiple reaction monitoring (MRM) scan mode. Two precursor-to-product ion transitions were simultaneously monitored at *m/z* 313.0–285.0, 313.0–269.0 for AFB_1_; *m/z* 315.0–259.0, 315.0–287.0 for AFB_2_; *m/z* 329.0–243.0, 329.0–215.0 for AFG_1_; *m/z* 331.0–245.0, 331.0–285.0 for AFG_2_. Instrumental data were collected using the Analyst^®^ Software version 1.6.2 with Schedule MRM TM Algorithm (AB Sciex, Toronto, ON, Canada).

Gas chromatography–mass spectrometry analyses were carried out using a gas chromatography-ISQ 3000 mass spectrometer (Thermo Scientific, San Jose, CA, USA) with a TG-5 MS capillary column (30 m × 0.25 mm i.d. 0.25 mm film thickness, Thermo Scientific, USA). Injection volume was 1.0 μL in split ratio of 10:1. Helium was used as carrier gas at 1.5 mL/min. Oven temperature was programmed as follows: initial temperature 50 °C for 3 min, raised to 75 °C (15 °C/min) for 0.5 min, raised to 110 °C (20 °C/min) for 1 min, raised to 130 °C (2 °C/min), raised to 140 °C (1 °C/min) and raised to 250 °C (5 °C/min) for 10 min. The temperature injector and MS transfer line were kept at 220 °C and 250 °C, respectively. Ionization was carried out in electron-impact ionization (EI) mode at 70 eV. The ion source temperature was set at 280 °C. The mass spectra were recorded within 40–500 amu in full scan mode.

### Response surface methodology for optimization of storage conditions

The reported optimum temperature and relative humidity for *A. flavus* growth are 20–40 °C and 80–95% (Hu et al. 2015), respectively. Similarly, the optimum temperature for the production of aflatoxin is 20–30 °C. Therefore, in this study, response surface methodology (RSM) with five levels (±1, ±α and one central point) and two-factor (temperature and humidity) central composite design (CCD) was used to optimize the storage conditions to prevent the infection of *A. oxyphylla* by *A. flavus*. The independent variables and their values for this study are shown in Table 1. The design consisted of 13 total experimental runs, which were analyzed using the Design expert 8.0.7.1 (Stat-Ease Inc. USA) statistical software package.

**Table 1:** Contents of AFs in *Alpinia oxyphylla* inoculated with *A. flavus* stored under different conditions

No.	Temperature (°C)	Humidity (%)	Content (µg/kg)				
			AFB_1_	AFB_2_	AFG_1_	AFG_2_	Total
A	22.93	82.20	<LOQ	ND	ND	ND	<LOQ
B	37.07	82.20	0.59	ND	ND	ND	0.59
C	22.93	92.80	1.77	ND	ND	ND	1.77
D	37.07	92.80	5.33	ND	ND	ND	5.33
E	20.00	87.50	<LOQ	ND	ND	ND	<LOQ
F	40.00	87.50	0.44	ND	ND	ND	0.44
G	30.00	80.00	<LOQ	ND	ND	ND	<LOQ
H	30.00	95.00	8.90	0.59	ND	ND	9.49
I	30.00	87.50	2.38	ND	ND	ND	2.38
J	30.00	87.50	2.48	ND	ND	ND	2.48
K	30.00	87.50	2.44	ND	ND	ND	2.44
L	30.00	87.50	2.53	ND	ND	ND	2.53
M	30.00	87.50	2.47	ND	ND	ND	2.47

Storage condition numbering in this paper is the same as shown in this table

*ND* not detected

Two hundred grams of *A. oxyphylla* samples were sterilized under a UV lamp for 2 h. Then the sterilized samples were divided into a control group (100 g) and an infection group (100 g). One milliliter of *A. flavus* spore suspension (10^6^ colony forming unit/mL) was added to the infection group samples, while 1 mL sterile water was added to the control group samples. The samples from the two groups were cultured under the same conditions for 10 days as a single experimental run. All the samples for 13 experimental runs were sterilized and dried for analysis.

### Determination of aflatoxin concentration by UPLC-MS/MS

At each time point, the cultured *A. oxyphylla* samples were prepared for aflatoxin (AFB_1_, AFB_2_, AFG_1_ and AFG_2_) analysis by UPLC-MS/MS according to the reported procedure and methods (Zhao et al. 2017). One gram of ground powder was placed into a 15-mL polycarbonate centrifuge tube, and was extracted with 4 mL of ACN-water-acetic acid (79:20:1, *v/v/v*). The tube was tightly capped and vortexed for 1.0 min, and then was placed into an ultrasonic bath at 40 °C for 20 min. The extracts were subsequently centrifuged for 15 min at 12,000 rpm. The supernatant was filtered through a 0.22-μm filter for UPLC-MS/MS analysis.

### Thin-layer chromatography of volatile oil

*Alpinia oxyphylla* volatile oils were prepared by hydrodistillation. Detailed methodology was as follows: 50 g of the ground powder was soaked in distilled water (tenfold volume) for 1.5 h, and extracted through steam distillation for 6 h. Then, the essential oils were dried over anhydrous sodium sulfate and sealed in an amber volumetric flask. Twenty-five microliters of the volatile oil was dissolved in 1.0 mL ethyl acetate for thin-layer chromatography (TLC) analysis.

Five microliters of the volatile oil solvents were separately applied on 20 × 20 cm chromatographic plates pre-coated with silica gel (Merck, Germany) as the stationary phase. The chromatograms were developed in a glass chamber containing cyclohexane-ethyl acetate (9:1, *v/v*) as the mobile phase. The plates were dried for 5 min and inspected under a UV lamp at 365 nm. TLC detection was performed in duplicate for all samples.

### GC–MS analysis

One gram of ground powder was extracted with 3 mL of ethyl acetate by sonication at room temperature for 30 min. The solution was adjusted to the original weight with ethyl acetate. The extracts were subsequently centrifuged at 5000 rmp for 10 min. Before being injected into the GC–MS, all solutions were filtered through 0.22-μm membrane filters.

### Determination of polysaccharide content

Polysaccharides were extracted from *A. oxyphylla* and detected according to the described protocol (Zhao et al. 2013). Half a gram of *A. oxyphylla* powder was weighed and placed into the extractor, then extracted by reflux with 25 mL petroleum ether (60–90 °C) and 25 mL 80% ethanol for 2 h, twice. Afterwards, the residue was extracted by reflux with 25 mL deionized water at 95 °C for 2 h, twice. Then, deionized water was added to the combined filtrate to achieve a final volume of 100 mL. One milliliter of the extract was transferred into a 10 mL test tube and 4 mL of anhydrous alcohol was added for precipitation overnight at 4 °C. The solution was centrifuged (6000 rpm, 10 min) to remove the supernatant. The precipitate was then dissolved in 500 mL deionized water for further analysis. One milliliter of sample solution was put into a test tube, then 1 mL phenol solution (5%) and 5 mL sulfuric acid were added and test tubes were shaken for 30 s. The test tube was heated by boiling in a water bath for 10 min, then was cooled to room temperature in an ice bath. The absorbance was measured at 484 nm. The polysaccharide content was calculated based on a standard curve of glucose.

## Results

### Aflatoxin detection in *A. oxyphylla*

All *A. oxyphylla* samples were cultured for 10 days under different storage conditions that were designed using CCD. Our findings show that mold growth in *A. oxyphylla* were different. As was shown in Additional file 1: Figure S1, *A. oxyphylla* samples were enveloped by hypha after storage in 30 °C and 95% humidity conditions. However, no hypha were observed on *A. oxyphylla* surfaces after storage in 20 °C and 87.5% humidity conditions. AFB_1_, AFB_2_, AFG_1_ and AFG_2_ levels were determined by optimized UPLC-MS/MS and the results are shown in Table 1 and Additional file 1: Figure S2. The linearity, and limits of detection (LOD) and quantification (LOQ) of the four investigated aflatoxins were determined and are shown in Additional file 1: Table S1. AFB_1_ was detected in all the samples, however aflatoxin content was below the LOQ (0.10 μg/kg) for sample No.1 (Temperature: 22.93 °C, Humidity: 82.2%), No.5 (Temperature: 20.0 °C, Humidity: 87.5%) and No. 7 (Temperature 30.0 °C, Humidity 80.0%). AFB_2_ was only detected in sample No. 8 (Temperature: 30.0 °C, Humidity: 95.0%) at a concentration of 0.59 µg/kg. The AFB_1_ content in sample No. 8 exceeded the limit set by Chinese Pharmacopoeia, and the total aflatoxin content (B_1_ + B_2_ + G_1_ + G_2_) was close to the maximum residue limits (MRL, 10 µg/kg). Additionally, the AFB_1_ concentration (5.33 µg/kg) in sample No.4 (Temperature: 37.07 °C, Humidity: 92.8%) also exceeded the MRL. For all the other samples, the content of AFB_1_ and total aflatoxins were within the acceptable range. AFG_1_ and AFG_2_ were not detected in any samples.

### Volatile oil yield

**Fig. 1:**
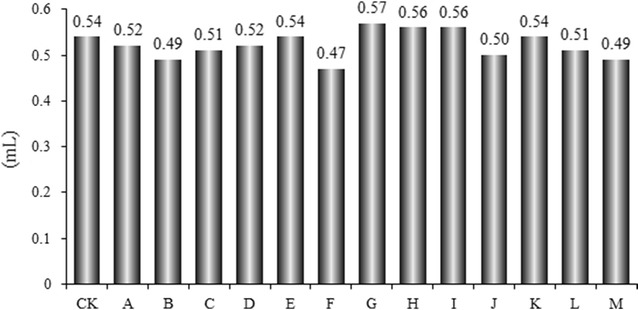
Effects of storage conditions on the yields of volatile oil in *A. oxyphylla*

Volatile oils are the main constituents of *A. oxyphylla*, and have been chosen as a quality control marker by the Chinese Pharmacopoeia (Chinese Pharmacopoeia Commission. 2015). Our results show that the volatile oil yields of *A. oxyphylla* ranged from 0.82 to 1.33 mL/100 g under normal storage conditions and were between 0.94 and 1.14 mL/100 g in experimental storage conditions (Miao et al. 2015). Mold growth caused small changes in volatile oil yield (Fig. 1) from *A. oxyphylla*, showing that mold infection could not influence the volatile oil content of this TCM.

### TLC analysis of volatile oil

**Fig. 2:**
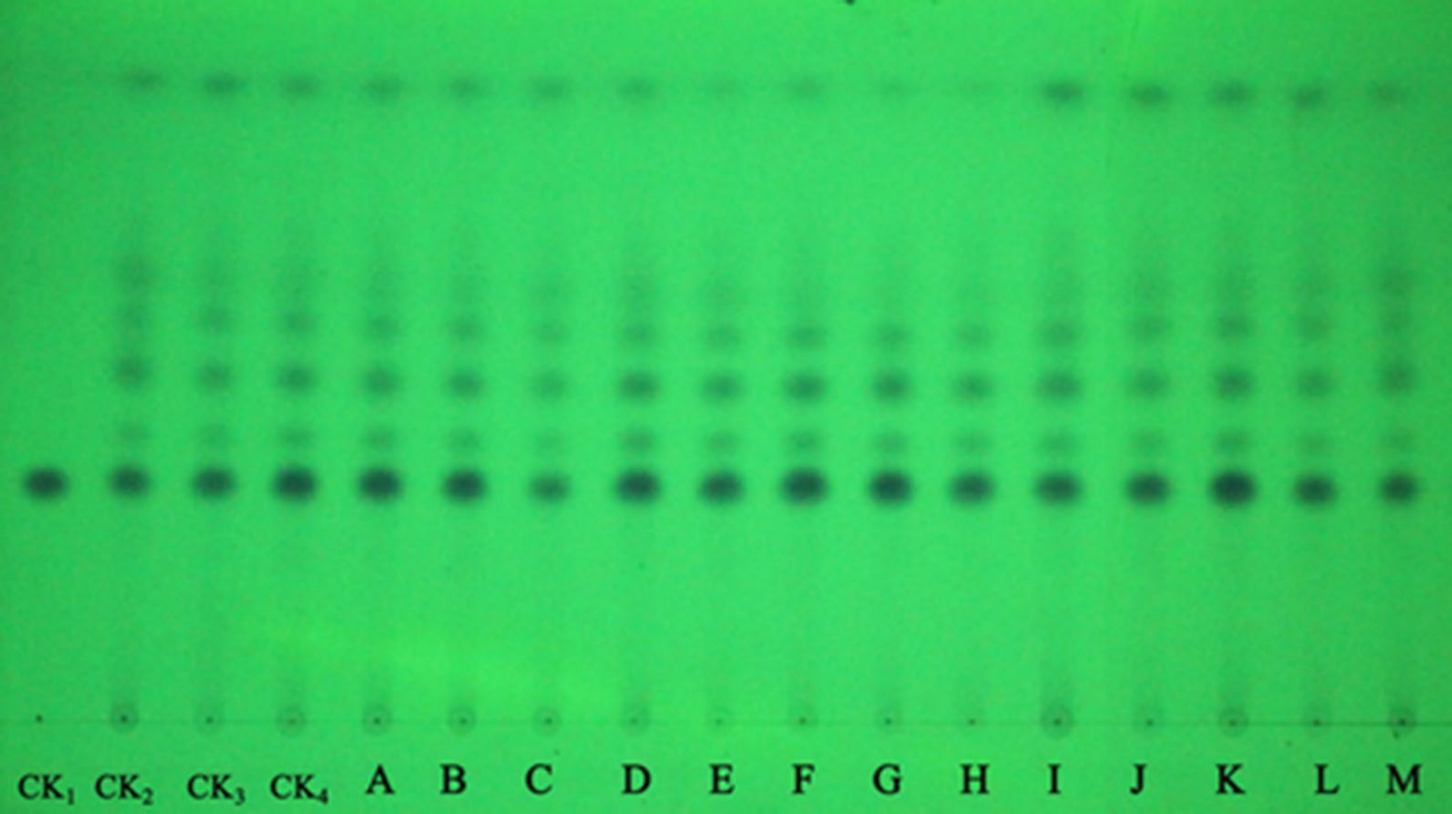
TLC of *A. oxyphylla* under different storage conditions. (CK1: Nootkatone; CK2, CK3, CK4, Sample stored at 4 °C)

Thin-layer chromatography is a very popular technique for the assessment of TCMs in Chinese Pharmacopoeia, due to the advantages of lower cost, less rigorous sample preparation, higher throughput, and easier visualization (Sowa and Subbaiah 2004). Although *A. flavus* infection caused small changes in the volatile oil yield of *A. oxyphylla*, the effect on its composition was unknown. Thus, we developed a convenient and cheap TLC method that targets only the essential oils. Photo documentation of TLC chromatograms is presented in Fig. 2. Overall, the visualized TLC spots in *A. oxyphylla* under different storage conditions were the same as the sample stored at 4 °C. Nootkatone was the most abundant volatile oil. TLC analysis confirmed that infection by *A. flavus* had no effect on the compositions of volatile oils of *A. oxyphylla*.

### GC–MS analysis

Gas chromatography–mass spectrometry is a powerful technique used for the analysis of volatile components, since it provides qualitative and quantitative data for complex mixtures, such as those usually present in herbs (Carrasco et al. 2015). To further confirm the effects of storage conditions on the volatile compounds in *A. oxyphylla*, we used the GC–MS method to analyze the ethyl acetate extract. Chromatograms of all samples are shown in Additional file 1: Figure S3. The volatile compositions of *A. oxyphylla* are reported in Table 2. A total of 37 volatile components were identified by GC–MS. Of these, nootkatone (10.23–12.30%), p-cymene (5.88–8.77%), alloaromadendrene (8.72–10.34%), aristolene epoxide (3.26–4.56%), α-selinene (1.87–2.43%), α-guaiene (1.85–2.31%), α-panasinsen (1.78–2.20%), caryophyllene oxide (1.85–2.49%), gingerol (2.04–2.94%) were found to be the principal chemical constituents. Similar results have been reported in other studies (Feng et al. 2015; Miao et al. 2015). As shown in Additional file 1: Figure S3 and Table 2, mold infection had small effects on the composition of volatile compounds in *A. oxyphylla*.

**Table 2:** Relative area (%) of common peaks in *A. oxyphylla* inoculated with *A. flavus* under different storage conditions

No	RT	Compounds	A	B	C	D	E	F	G	H	I	J	K	L	M	CK
1	5.52	α-Pinene	0.38	0.38	0.29	0.39	0.37	0.41	0.33	0.27	0.36	0.27	0.38	0.36	0.34	0.41
2	6.25	β-Pinene	0.44	0.50	0.41	0.49	0.33	0.34	0.29	0.52	0.35	0.41	0.46	0.36	0.40	0.40
3	6.88	p-Cymene	8.77	7.12	6.19	8.31	8.14	6.77	5.94	6.98	6.52	6.34	6.54	5.88	6.61	7.71
4	7.31	γ-Terpinene	1.03	1.00	0.88	1.04	0.84	0.74	0.82	0.74	0.63	0.75	0.72	0.86	0.84	0.65
5	7.71	Undecane	1.20	1.31	1.23	1.08	1.54	1.50	1.04	1.25	1.39	1.67	1.44	1.68	1.78	1.05
6	7.88	Terpinen-4-ol	0.43	0.54	0.39	0.48	0.54	0.63	0.53	0.40	0.40	0.51	0.45	0.47	0.64	0.53
7	8.68	Isopinocarveol	0.32	0.33	0.29	0.26	0.38	0.48	0.27	0.30	0.34	0.33	0.28	0.30	0.32	0.25
8	9.52	Dodecane	0.89	0.90	0.92	0.84	0.81	0.91	0.75	0.90	1.06	1.21	1.00	1.18	1.02	0.91
9	11.37	Tridecane	0.52	0.44	0.40	0.46	0.36	0.35	0.48	0.28	0.39	0.36	0.29	0.28	0.29	0.38
10	14.5	Copaene	1.23	1.20	1.17	1.20	1.38	1.26	1.14	1.29	1.13	0.83	0.80	1.15	1.30	1.35
12	17.19	α-selinene	2.13	1.97	2.22	2.44	1.87	1.89	2.04	2.37	2.24	2.00	1.90	2.43	1.96	2.05
13	17.27	β-ylangenen	0.74	0.69	0.78	0.75	0.69	0.76	0.72	0.75	0.79	0.69	0.64	0.70	0.75	0.69
14	17.52	Humulene	0.54	0.60	0.47	0.49	0.60	0.48	0.52	0.64	0.64	0.54	0.56	0.56	0.58	0.50
15	17.69	Aromandendrene	0.98	0.91	1.07	1.07	0.90	0.98	1.00	0.88	1.12	0.90	0.87	0.89	1.01	1.03
16	18.71	α-Guaiene	2.11	1.88	1.98	2.20	2.19	1.78	2.02	2.31	2.31	1.98	1.98	2.15	1.85	1.98
17	19.07	Alloaromadendrene	10.06	9.35	9.15	10.34	9.77	10.22	9.85	9.72	9.65	9.17	9.39	8.72	9.00	8.94
18	19.53	α-Farnesene	0.27	0.47	0.36	0.38	0.38	0.31	0.42	0.27	0.31	0.32	0.32	0.26	0.30	0.29
19	19.71	β-Bisabolene	0.32	0.32	0.36	0.31	0.36	0.30	0.32	0.27	0.28	0.33	0.38	0.27	0.26	0.32
20	20.13	cubedol	0.62	0.59	0.78	0.56	0.68	0.55	0.61	0.57	0.57	0.61	0.70	0.70	0.66	0.59
21	20.31	(-)-α-Panasinsen	2.20	1.89	2.01	1.78	2.60	1.80	2.20	1.92	2.10	2.02	1.96	1.95	2.05	2.27
22	22.87	trans-Z-α-Bisalolene epoxide	0.47	0.54	0.58	0.51	0.48	0.47	0.46	0.50	0.56	0.43	0.47	0.43	0.46	0.42
23	23.55	Caryophyllene oxide	2.11	2.21	2.39	1.85	2.24	2.29	1.93	1.93	2.13	2.37	2.49	2.22	2.20	1.95
24	25.18	Humulene 1,2-opoxide	2.80	2.49	2.83	2.23	3.73	2.91	2.59	2.67	2.16	3.07	3.20	2.96	3.13	2.74
25	26.23	cis-Z-α-Bisalolene epoxide	0.40	0.34	0.48	0.46	0.48	0.39	0.39	0.47	0.50	0.44	0.50	0.43	0.48	0.40
26	28.17	Caryophyllene oxide	1.30	1.09	1.17	1.10	1.33	1.10	1.04	1.21	1.23	1.18	1.23	1.30	1.19	1.19
27	28.47	γ-Elemene	0.94	0.92	0.89	1.03	0.95	0.80	0.83	0.86	0.88	0.89	0.94	0.94	0.93	0.83
28	28.59	ledol	0.51	0.58	0.65	0.50	0.62	0.54	0.53	0.66	0.58	0.63	0.58	0.60	0.60	0.69
29	29.1	Isoaromadendrene epoxide	1.49	1.45	1.39	1.19	1.44	1.45	1.27	1.47	1.68	1.29	1.51	1.60	1.64	1.37
30	30.25	Aristolene epoxide	3.27	3.26	4.13	4.00	4.48	4.00	3.62	3.76	4.04	4.21	4.37	4.56	4.16	4.21
31	31.04	Calarene epoxide	0.80	0.84	0.93	0.83	1.06	0.87	0.81	0.86	0.80	0.94	0.98	0.83	0.91	0.99
32	31.47	cis-Z-α-Bisabolene epoxide	1.12	1.03	1.23	1.18	1.47	1.28	1.12	1.27	1.35	1.20	1.20	1.27	1.33	1.23
33	33.32	4,5-di-epi-aristolochene	0.90	0.99	1.05	0.85	1.12	0.85	0.84	0.86	0.87	0.98	1.03	1.12	1.09	1.00
34	34.43	Nootkatone	10.31	10.55	10.99	10.35	10.23	11.13	12.30	10.34	11.31	10.49	10.48	11.66	10.59	10.66
35	34.81	Longipinocarvone	0.95	1.03	0.96	1.05	1.04	0.90	0.92	1.08	0.85	0.99	1.02	1.09	1.01	1.04
36	36.47	Longifolenaldehyde	0.93	1.01	1.18	1.23	0.90	1.15	0.95	1.70	1.80	1.07	1.05	1.11	1.02	1.20
37	51.88	Gingerol	2.39	2.40	2.84	2.78	2.61	2.34	2.52	2.04	2.23	2.97	2.57	2.17	2.94	2.13

### Analysis of polysaccharides

**Fig. 3:**
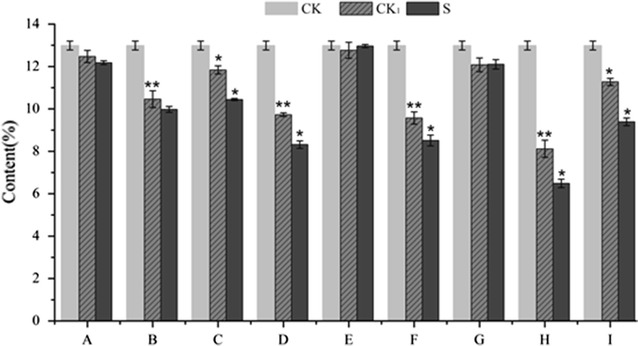
Effects of storage condition on the content of polysaccharide of *A. oxyphylla* inoculated with *A. flavus* (* means significant at 0.05 level; ** means significant at 0.01 level; CK, Sample stored at 4 °C; CK_1_, Accompanying control; S, Sample under different storage conditions)

To quantify the polysaccharide content of *A. oxyphylla,* we used the phenol–sulfuric acid method, and used glucose as the standard monosaccharide. The calibration curve was linear within the range of 2.05–65.60 μg/mL, with a correlation coefficient of 0.999. We found that the polysaccharide content of *A. oxyphylla* was 12.91% (Fig. 3). After co-incubation with *A. flavus* for 10 days, the polysaccharide content reduced remarkably, especially for the sample stored at 30.00 °C and 95.00% humidity. In addition, the polysaccharide content was significantly lower than control samples when *A. oxyphylla* was stored under the following conditions: 37.07 °C, 92.80% humidity (D); 40 °C, 87.5% humidity (F); and 30.00 °C, 87.5% humidity (I). Furthermore, under these conditions, *A. flavus* grew well and the aflatoxin concentrations were very high. It was worth noting that the polysaccharide content of several control samples (except A, E and G) under the same storage conditions was lower compared to samples stored in the refrigerator (4 °C).

## Discussion

Stored *A. oxyphylla* is a man-made ecosystem in which quality and nutritive changes occur because of interactions between physical, chemical and biological factors. Fungal spoilage and mycotoxin contamination are a major concern. If storage conditions are poorly managed, *Aspergillus* species can infect *A. oxyphylla* and mycotoxin contamination can occur (Chulze 2010). High temperatures and high humidity are two important factors leading to mold growth and aflatoxin contamination in the storage of cereals, feeds and herbs (Müller and Basedow 2007). *A. oxyphylla* is widely cultivated in tropical and subtropical regions, particularly in the Hainan province, which is located at 18°10′–20°10′N latitude and 108°37′–111°03′E longitude. There, the climate is warm, with plentiful rain and high humidity. Consequently, *A. oxyphylla* fruit may be more sensitive to mildew during storage such locations. In our previous study, none of the samples we tested had aflatoxin content exceeding the regulated maximum amount allowed (Zhao et al. 2017). However, high incidence and levels of AF contamination in *A. oxyphylla* has been reported (Zha et al. 2014; Zhang et al. 2008). Improved storage conditions to prevent *A. oxyphylla* spoilage and reduce aflatoxin contamination are recommended.

According to the results described above, AFB_1_ content should increase with increasing the temperature given the same humidity during storage, and vice verse. Our results showed that temperature and humidity played an important role in the growth of the *A. flavus* and the production of aflatoxins. After analysis using RSM, we concluded that the growth of *A. flavus* and production of aflatoxins could be minimized by maintaining the temperature below 25 °C and the humidity below 85%. Therefore, these conditions can be recommended as the best storage conditions for preventing the infection of the *A. oxyphylla* sample by *A. flavus*. Liu found that *Areca catechu* was not susceptible to mildew infection or toxin production in environments with humidity below 90% and temperature below 25 °C (Liu et al. 2015). Furthermore, the best storage conditions for Radix Astragali to avoid *A. flavus* contamination were temperature and humidity below 20 °C and 85% (Hu et al. 2015). These results may indicate that the optimal storage conditions of medicinal materials were related to TCMs.

Volatile oil is one of the main components of *A. oxyphylla,* and is used as a marker compound to assess the quality of *A. oxyphylla* in the Chinese pharmacopoeia (Chinese Pharmacopoeia Commission 2015). The composition of volatile compounds in *A. oxyphylla* infected by *A. flavus* was similar to the control samples, which might be related to the properties of these components. *A. flavus* appears to mainly rely on polysaccharides, proteins, and fatty acids in *A. oxyphylla* as its source of nutrition, rather than the volatile components in volatile oils. These results have been confirmed by Prakash et al. (2015). The chemical variability of volatile oils due to variable ecological and geographical conditions, plant species, harvest time and extraction methodology were the major issues for their application as natural preservatives.

Polysaccharides are natural biological macromolecules that are composed of monosaccharide units bound together by glycosidic linkages (Liu et al. 2016). Polysaccharides are widely found in plants, which use them as energy storage molecules, structural components and protective substances (Le Floch et al. 2015). As a kind of carbohydrate, polysaccharides can provide a carbon source for the growth of fungi (Coutinho et al. 2009). As the main bioactive component of *A. oxyphylla*, polysaccharides can be used to systematically evaluate *A. oxyphylla* quality. Therefore, it is important to accurately analyze polysaccharides in *A. oxyphylla* under different storage conditions after infection with *A. flavus. Aspergilli* can use a wide variety of carbon compounds as a carbon source for its growth. These compounds include polysaccharides, oligo- and disaccharides, hexoses, pentoses, organic acids, aromatic compounds, alcohols, polyols, and fatty acids (Norihiro Tsukagoshi and Masashi 2001). Several species in the *Aspergillus* genus, including *A. flavus,* can produce and utilize enzymes that degrade polysaccharides (de Vries 2003). Therefore, polysaccharides could be used as the carbon source for the growth of *A. flavus*, which may be the main reason for the decrease in polysaccharides we observed in the *A. oxyphylla* samples after *A. flavus* infection. Furthermore, we found that the polysaccharide content of control samples (except A, E and G) under the tested storage conditions was lower compared to samples stored in the refrigerator (4 °C). This was especially true for storage temperatures above 30 °C. Under these conditions, enzymatic activity would promote polysaccharide degradation. Additionally, as carbohydrates are oxidized during respiration, the heat produced by respiration will accelerate polysaccharide loss. Certainly, the role of water in the degradation of polysaccharides cannot be ignored. These results confirm that storage conditions have an important influence on the quality of *A. oxyphylla*.

In brief, TCMs undergo numerous physical, chemical and microbiological changes, including mold infection, if they are stored under improper conditions. In this paper, how *A. oxyphylla’*s storage conditions impact its quality and mildew growth was systematically studied. After mold infection, the volatile oil composition and the volatile components of *A. oxyphylla* were unaffected, but the polysaccharide content was reduced remarkably and mycotoxins were found, which would affect the quality and safety of *A. oxyphylla*. The crucial storage factors (including temperature and humidity) for preventing mold growth in *A. oxyphylla* were analyzed using RSM. Storing dry *A. oxyphylla* at temperatures below 25 °C and humidity below 85% could effectively inhibit the development of *A. flavus* mold infection. Mildew infections caused by other toxic fungi and the resulting changes to bioactive constituents in *A. oxyphylla* will be explored in future experiments.

## Additional file

**Additional file 1: Figure S1.** *A. oxyphylla* inoculated with *A. flavus* conidial suspension and cultured for 10 days. **Figure S2.** UPLC-MS/MS MRM chromatograms of 4 aflatoxins. **Figure S3.** GC–MS chromatograms of all the samples inoculated with *A. flavus* under different storage conditions. **Table S1.** Linearity, LODs, LOQs, of the 4 investigated mycotoxins.

## Abbreviations

*A. oxyphylla*: *Alpinia oxyphylla*
*A. flavus*: *Aspergillus flavus*
TCMs: traditional Chinese medicines
AFs: aflatoxins
GC–MS: gas chromatography–mass spectrometry
UPLC-MS/MS: ultra-performance liquid chromatography coupled with tandem mass spectrometry
ESI: electrospray ionization
TLC: thin-layer chromatography
RSM: response surface methodology
CCD: central composite design
