# Supplementary material for: Quality evaluation of Alpinia oxyphylla after Aspergillus flavus infection for storage conditions optimization
Source: AMB Express. 2017 Jul 11;7:151. doi: 10.1186/s13568-017-0450-x (PMC5503849; doi:10.1186/s13568-017-0450-x)
Supplement: Supplementary file 1 — Additional file 1: Figure S1. A. oxyphylla inoculated with A. flavus conidial suspension and cultured for 10 days. Figure S2. UPLC-MS/MS MRM chromatograms of 4 aflatoxins. Figure S3. GC–MS chromatograms of all the samples inoculated with A. flavus under different storage conditions. Table S1. Linearity, LODs, LOQs, of the 4 investigated mycotoxins. [file 13568_2017_450_MOESM1_ESM.docx]

**Supplementary Materials**

**Quality evaluation of *Alpinia oxyphylla* after *Aspergillus flavus* infection for storage conditions optimization**

Xiangsheng Zhao ^1^, Jianhe Wei ^1,2^, Yakui Zhou ^1^,Weijun Kong ^2🖂^, Meihua Yang ^1,2🖂^

^1^*Hainan Branch Institute of Medicinal Plant Development, Chinese Academy of Medical Sciences* & *Peking Union Medical College, Haikou 571100, China;*

^2^*Institute of Medicinal Plant Development, Chinese Academy of Medical Sciences & Peking Union Medical College, Beijing 100193, China*；


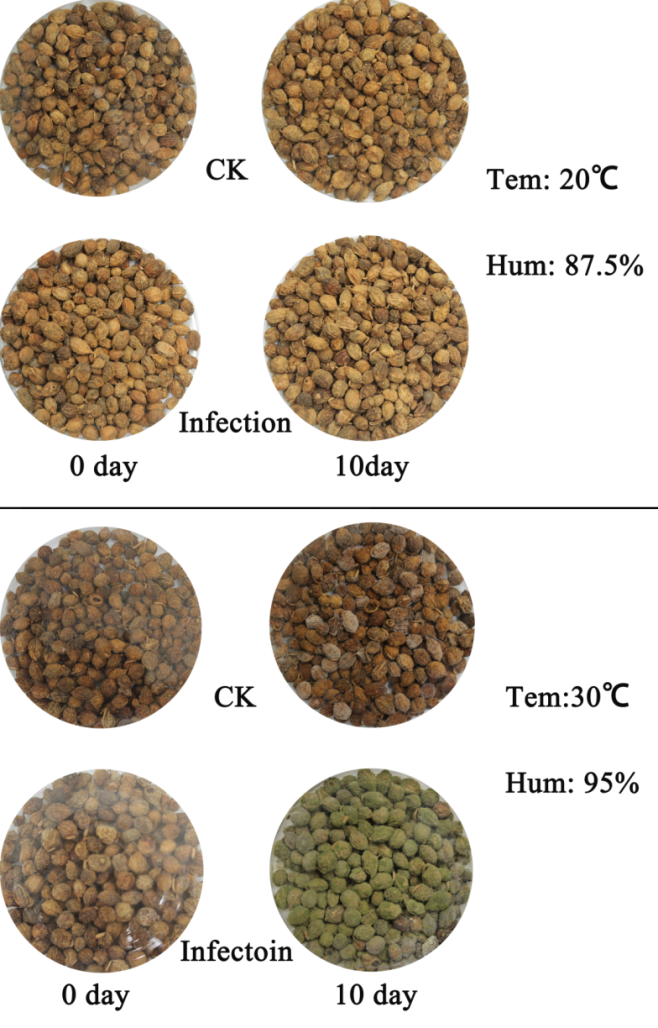


**Fig. S1.** *A. oxyphylla* inoculated with *A. flavus* conidial suspension and cultured for 10 days.


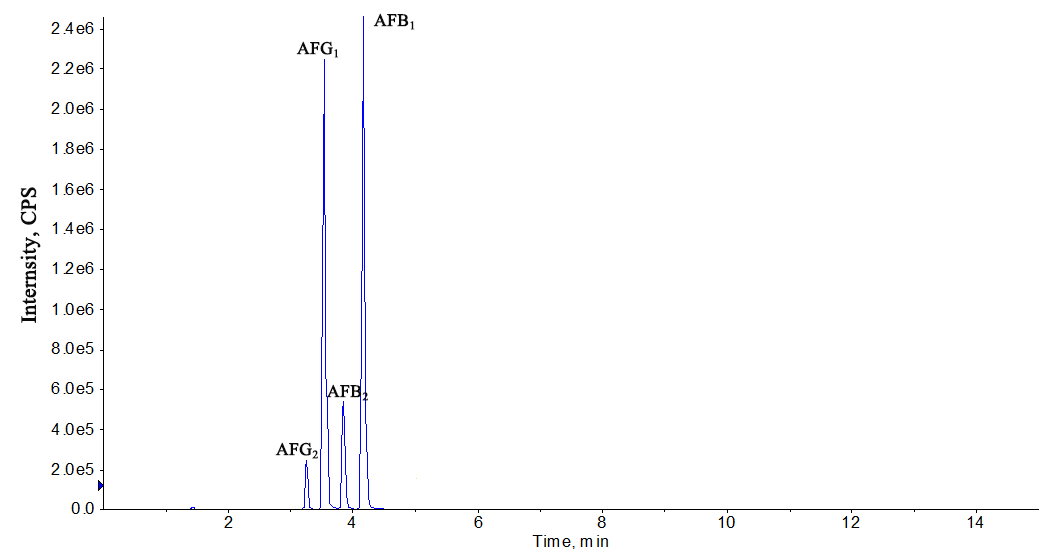


**Fig. S2.** UPLC-MS/MS MRM chromatograms of 4 aflatoxins.


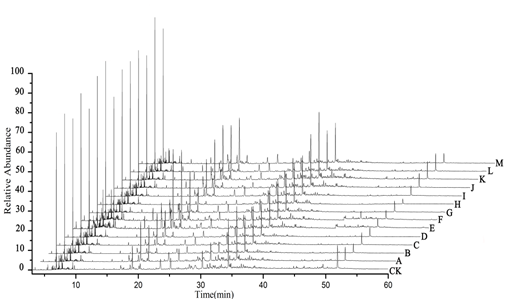


**Fig.S3.** GC-MS chromatograms of all the samples inoculated with *A. flavus* under different storage conditions

Table S1 Linearity, LODs, LOQs, of the 4 investigated mycotoxins.

| Analytes | Linear equation | *R^2^* | Range  (μg/kg) | LOD  (μg/kg) | LOQ  (μg/kg) |
| --- | --- | --- | --- | --- | --- |
|  |  |  |  |  |  |
| AFG_2_ | *Y* = 16556*X* - 2352.7 | 0.9985 | 0.10-25 | 0.03 | 0.10 |
| AFG_1_ | *Y* = 48742*X* - 23488 | 0.9969 | 0.15-50 | 0.05 | 0.15 |
| AFB_2_ | *Y* = 28862*X* + 648.1 | 0.9958 | 0.20-25 | 0.05 | 0.20 |
| AFB_1_ | *Y* = 42974*X* + 4681.2 | 0.9982 | 0.10-50 | 0.03 | 0.10 |
